# Nutritional approaches to counter performance constraints in high‐level sports competition

**DOI:** 10.1113/EP088188

**Published:** 2021-11-26

**Authors:** Louise M. Burke

**Affiliations:** ^1^ Exercise and Nutrition Research Program Mary MacKillop Institute for Health Research Australian Catholic University Melbourne Australia

**Keywords:** competition nutrition, glycogen loading, hydration, mouth sensing

## Abstract

**New Findings:**

- **What is the topic of this review?**
  The nutritional strategies that athletes use during competition events to optimize performance and the reasons they use them.
- **What advances does it highlight?**
  A range of nutritional strategies can be used by competitive athletes, alone or in combination, to address various event‐specific factors that constrain event performance. Evidence for such practices is constantly evolving but must be combined with understanding of the complexities of real‐life sport for optimal implementation.

**Abstract:**

High‐performance athletes share a common goal despite the unique nature of their sport: to pace or manage their performance to achieve the highest sustainable outputs over the duration of the event. Periodic or sustained decline in the optimal performance of event tasks, involves an interplay between central and peripheral phenomena that can often be reduced or delayed in onset by nutritional strategies. Contemporary nutrition practices undertaken before, during or between events include strategies to ensure the availability of limited muscle fuel stores. This includes creatine supplementation to increase muscle phosphocreatine content and consideration of the type, amount and timing of dietary carbohydrate intake to optimize muscle and liver glycogen stores or to provide additional exogenous substrate. Although there is interest in ketogenic low‐carbohydrate high‐fat diets and exogenous ketone supplements to provide alternative fuels to spare muscle carbohydrate use, present evidence suggests a limited utility of these strategies. Mouth sensing of a range of food tastants (e.g., carbohydrate, quinine, menthol, caffeine, fluid, acetic acid) may provide a central nervous system derived boost to sports performance. Finally, despite decades of research on hypohydration and exercise capacity, there is still contention around their effect on sports performance and the best guidance around hydration for sporting events. A unifying model proposes that some scenarios require personalized fluid plans while others might be managed by an ad hoc approach (ad libitum or thirst‐driven drinking) to fluid intake.

## 1 INTRODUCTION

**FIGURE 1:**
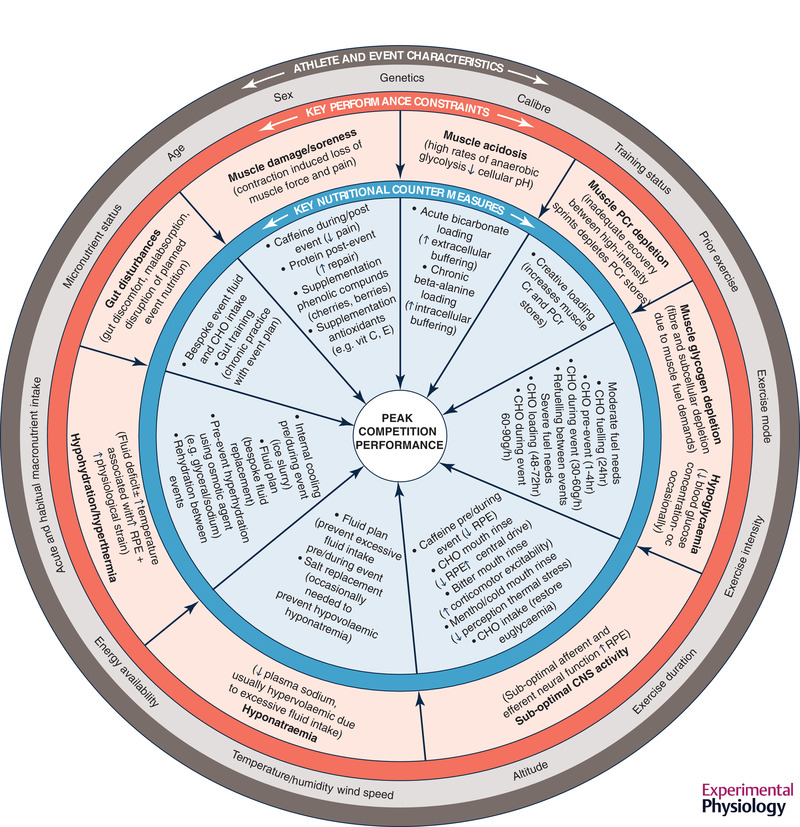
A range of factors related to the exercise task, the athlete and the environment interact to create physiological conditions that can constrain important attributes of competition performance. In many cases, key performance constraints can be addressed by nutritional strategies undertaken before, during and between events to reduce or delay the onset of the performance decline. CHO, carbohydrate; CNS, central nervous system; Cr, creatine; PCr, phosphocreatine; RPE, ratings of perceived exertion

Sports competitions range from seconds to weeks, in a variety of physical and climatic environments, and with variety in the number and characteristics of discrete participants, the rules of participation, and the procedures for determining the outcomes of the event. Despite the unique features of their sport, all athletes share a common goal: to pace or manage their performance to achieve the highest sustainable outputs or speeds, with technical and tactical proficiency, over the duration of the event. Although many athletes target competitions to showcase maximum performance to break world records or achieve personal bests, for others, success simply involves a performance that is superior to those of other competitors. Regardless, competition success is commonly determined by the athlete's ability to manage a decline in some performance metrics, intermittently and/or towards the end of the event. A periodic or sustained decline in the ability to optimally perform the tasks required of the specific event involves interplay between central and peripheral phenomena that constrain power or force production, increase ratings of fatigue/pain or reduce skill and cognitive abilities. Common causes of these physiological constraints include depletion of the substrate(s) needed to support the muscle turnover of adenosine triphosphate (ATP) or changes in body homeostasis (see Figure 1). Meanwhile, contemporary competition nutrition practices undertaken before and during an event can reduce or delay the onset of specific factors that would otherwise acutely constrain performance in a targeted event (Burke & Hawley, 2018). This review will summarize recent updates in our knowledge and practice around several of the themes included in Figure 1.

## 2 DEVELOPING STRATEGIES TO ADDRESS PHYSIOLOGICAL CONSTRAINTS TO COMPETITION PERFORMANCE

The model of competition nutrition as a targeted approach to the physiological constraints of exercise explains many of the guidelines included in recent expert statements on sports nutrition (Burke, Castell, et al., 2019; Collins et al., 2021; Thomas et al., 2016) and observed in real‐life sporting events (Fordyce, 2018). Furthermore, it paves the way for the development or refinement of new strategies once mechanisms to explain causes of performance decrement are identified and an evidence‐based countermeasure proposed. Sometimes these strategies are developed in the laboratory from a first principles knowledge of biochemistry and exercise physiology. An exemplar of this is beetroot juice consumption, as a means of nitrate supplementation, which was derived from laboratory‐tested hypotheses that it might provide an alternative pathway for Nitric Oxide production, aiding muscle contractility and more economical oxygen utilization, especially under hypoxic and acidotic conditions in which the primary arginine pathway is disabled (Jones et al., 2018). Alternatively, other strategies represent the scientific validation of practices developed through trial and error by athletes and coaches. For example, it was only in the early 2000s that sports scientists proved that the consumption of small amounts of caffeine in the final portion of an endurance race, as observed among road cyclists and runners and often achieved via the intake of flattened cola beverages, achieved the same performance benefit as the traditional laboratory‐derived protocol of consuming large caffeine doses an hour prior to exercise (Cox et al., 2002). Nevertheless, before examining our current knowledge of nutritional factors that counter performance constraints in sports competition, it is important to recognize the challenges of developing and implementing solutions that can be applied to elite athletes in real world settings.

First, in many sports, the dynamic and changing nature of competition makes it difficult to predict and address the actual limitations to performance in any single event. For example, every match in a team or racket sport is literally and figuratively ‘a new ball game’, requiring athletes to prepare for each competition in anticipation of the likely, rather than certain, risk factors that might interfere with optimal performance. Even in sports with a pre‐determined workload (e.g., a marathon or a 1500 m swim), the overlay of the environment (heat, humidity, wind, altitude, terrain, playing surface), the competition schedule (time of event, period between heats/finals, etc.) and the need for travel (food environment, circadian effects) adds further physiological and practical challenges. Therefore, there is always a need to evolve new and bespoke strategies for different versions of the same event.

Second, in most sports, performance is limited by a number of interdependent factors, which need to be addressed by a coordinated plan. The interplay between performance‐limiting factors or the strategies used as countermeasures could involve redundancy, amplification, attenuation or competition between effects (Burke, 2017). Inadequate attention has been paid to the net outcome of combining nutrition strategies that are independently valuable, despite evidence of important interactions. For example, the interaction of caffeine supplementation and carbohydrate intake during endurance events reduces the magnitude of effect of the former (Conger et al., 2011), but the individual benefits of fluid and CHO replacement on performance in the heat appear to be additive (Below et al., 1995). Meanwhile, bicarbonate supplementation may negate the benefits of caffeine use during sustained maximal exercise events due to gastrointestinal side effects (Grgic, 2020). Whether strategies can be repeated within a short timeframe for sports involving multi‐stage, multi‐day or multi‐event competitions is also pertinent. Since it is impractical to investigate the effects of the many permutations and combinations of evidence‐based nutrition strategies using conventional research design (Burke, 2017; Burke & Peeling, 2018), athletes are often reliant on trial and error rather than answers from the peer‐reviewed literature.

Third, even when beneficial nutritional strategies can be identified, the implementation of these strategies before, during and between events is dependent on the rules, logistical considerations and cultures that exist in sport (Garth & Burke, 2013). While some sports offer opportunities for regular access to foods and fluids during competition, conditions in other events prevent optimal practice. Therefore, the real world includes scenarios in which successful athletes achieve substantial nutritional support (e.g., the road cyclist, aided by feedzones and the ferrying of nutritional supplies by *domestique* teammates, who consumed 500 g CHO during a 5‐h cycling stage towards a daily CHO intake of 18 g kg^−1^ body mass (BM); Fordyce, 2018) while other events present a mismatch between requirements and opportunities for intake (e.g., the winner of a hot weather marathon incurring fluid losses equivalent to 10% BM due to the practical challenge of drinking while running at ∼21 km h^−1^ from aid stations placed at every 5 km; Beis et al., 2012). Notwithstanding the athletes’ villages and their sophisticated catering arrangements at many premier events (Pelly & Parker Simmons, 2019), the global nature of elite sport challenges athletes to achieve their competition practices in foreign environments, sometimes against a backdrop of reduced food availability, atypical food choices and customs, and sub‐optimal food hygiene (Halson et al., 2019).

Finally, the importance of the psychological aspects of competition nutrition strategies should be respected and incorporated into evidence‐based practices. Indeed, at least part of the benefit of competition nutrition strategies (even when they fall outside guidelines) comes from psychological factors ranging from familiarity to a placebo or expectancy effect (Raglin et al., 2020). This review will illustrate the interaction of these caveats with some of the competition nutrition strategies that provide a counter‐measure to performance‐limiting factors (Figure 1), focusing on areas of new and emerging information around three themes: fuel availability, central nervous system (CNS) effects and hydration. For updated information on evidence‐based performance aids, the reader is directed to other reviews (Peeling et al., 2018).

## 3 COMBATING COMPETITION PERFORMANCE DECLINE BY SUPPORTING FUEL AVAILABILITY

The energetics of sporting events may require up to a 100‐fold increase in ATP turnover. Training enhances the metabolic flexibility of the muscle, increasing the size of substrate pools and fine‐tuning the regulation and integration of the various metabolic pathways by which ATP is resynthesized (Hawley et al., 2018). The reader is referred to several contemporary reviews of the metabolic pathways that, in various combinations, contribute to the fuel demands of all exercise tasks (Hargreaves & Spriet, 2020; Hawley et al., 2014). However, a simplistic model identifies the importance of the rapid activation of metabolic pathways for ATP regeneration during short‐term (<30 s) sprints, primarily through substrate‐level phosphorylation or ‘anaerobic’/oxygen‐independent metabolism: the breakdown of phosphocreatine (PCr) and the conversion of muscle glycogen to lactate. Meanwhile, sporting activities lasting several minutes to several hours, whether ‘steady‐state’ or involving intermittent high‐intensity bursts, are principally fuelled by the oxidation of intramuscular glycogen and lipids (‘aerobic’ metabolism), with the mobilization of extra‐muscular substrates (plasma glucose from liver and gut, and free fatty acids (FA) released from adipocytes) becoming more important as exercise duration increases. A range of competition nutrition strategies address the relative lack of substrate for key pathways.

### 3.1 Creatine supplementation to boost PCr stores

Although creatine/PCr plays a large number of roles within the muscle and CNS (Marques & Wyse, 2019) and other body tissues (Bonilla et al., 2021), the bioenergetic roles of PCr receive most attention. As well as shuttling intracellular energy from the mitochondria to the cytosol, it has a limiting role within the skeletal muscle cell as an energy reservoir that rapidly responds to a sudden increase in energy turnover, restoring cellular ATP without the need for oxygen (Wallimann et al., 2011). As such, PCr provides a critical substrate for single and intermittently repeated sprints, with the gradual decline in force/power/speed during repeated high‐intensity work bouts being associated with a failure to fully restore PCr concentrations during the recovery interval between bouts (Bogdanis et al., 1996). In 1992, a scientific publication summarizing increases in muscle creatine/PCr following oral intake of creatine monohydrate (Harris et al., 1992) coincided with revelations that such a regimen had been undertaken by successful sprinters at the Barcelona Olympic Games, introducing the sporting world to creatine supplementation. Three decades later, creatine has become one of the highest selling and most researched sports supplements, with potential applications to a range of clinical conditions (Gualano et al., 2012) as well as sports performance. Despite the marketing of exotic creatine compounds, creatine monohydrate is the main form of supplemental creatine, and can be safely and effectively taken as an acute loading protocol (5 d @ 20 g day^−1^ in split doses) or as a longer maintenance dose (4 weeks @ 3 g day^−1^) to achieve a typical increase of ∼20% in muscle PCr and creatine stores (Kreider et al., 2017). Muscle creatine uptake is enhanced when creatine supplements are co‐ingested with CHO or protein (Steenge et al., 2000) and creatine supplementation may be more effective in vegetarian athletes who lack a dietary creatine source and are reliant on endogenous production that normally accounts for about half the muscle creatine content (Kaviani et al., 2020).

Increased muscle PCr/creatine content can attenuate the decline in repeated sprint ability when high‐intensity exercise (<30 s) is interspersed with short recovery intervals (e.g., ∼1 min), which fail to allow complete restoration of PCr between bouts (Greenhaff et al., 1993). Creatine supplements are therefore most often positioned as a training aid, where enhanced energetics may allow the athlete to train harder or with higher quality during interval or resistance workouts, or to benefit from other mechanisms such as upregulation of gene expression and protein synthesis mediated via changes in cellular osmolality due to the storage of creatine in combination with water (Safdar et al., 2008). Nevertheless, it may directly enhance competition outcomes in intermittent sprint events such as team and racket sports, if the energetics of game characteristics mimic the work:rest ratios that it supports (Mielgo‐Ayuso, Calleja‐Gonzalez, Marques‐Jimenez, et al., 2019) or to address the integration of stochastic sprint efforts within an endurance event (Tomcik et al., 2018). It should be noted, however, that very few of the hundreds of studies of creatine supplementation and sports/exercise performance have been conducted in real‐world protocols or events. Newer areas of interest around acute creatine supplementation and competition performance include the role of enhanced brain creatine stores on skill and cognitive function in stressful or sub‐optimal conditions (e.g., when the athlete is sleep‐deprived), as well as neuroprotection against mild brain traumatic injuries (Roschel et al., 2021).

### 3.2 Strategies to enhance muscle CHO availability

High‐intensity exercise underpins success in the majority of competitive sports, whether in the form of brief events conducted at intensities above ${\dot{V}}_{O_{2}\mathrm{peak}}$, endurance exercise sustained in highly aerobic domains (80–100% ${\dot{V}}_{O_{2}\mathrm{peak}}$) or events involving combinations of both, such as intermittent team/racket sports or stochastic endurance events including critical ‘breakaway’ pieces interspersed between more moderate intensity workloads (e.g., road cycling). The features and regulation of CHO metabolism are elegantly suited to fuel such events, since CHO can be used for both aerobic and oxygen‐independent production of ATP, with rapid activation of these pathways being able to meet fuel needs across the whole range of power demands and the transitions between them (Hargreaves & Spriet, 2020). In addition, CHO oxidation provides more economical generation of ATP than does lipid oxidation, with the 5–7% greater ATP yield per litre of oxygen consumed (Krogh & Lindhard, 1920) becoming important at high‐intensity domains (Burke, 2021).

**FIGURE 2:**
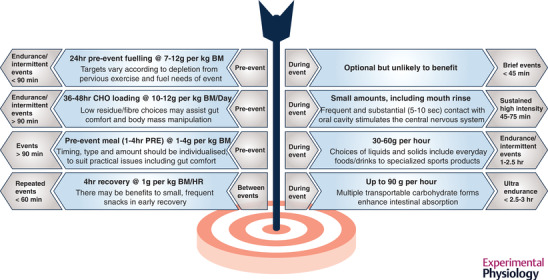
Guidelines for carbohydrate intake before, during and between sporting events to maintain adequate fuel availability for the muscle and central nervous system to optimize competition performance (Thomas et al., 2016). BM, body mass

CHO fuels for the muscle include its own glycogen stores, blood glucose derived from liver glycogenolysis and gluconeogenesis and exogenous CHO supplied from the gut via the intake of CHO immediately before and during the event. The application of the muscle biopsy technique to sports science in the 1960s established that the depletion of muscle glycogen during exercise is associated with fatigue (Bergstrom et al., 1967) and precipitated a fertile theme in sports nutrition research around strategies to match the labile and relatively limited body CHO stores to the fuel requirements of exercise (Burke et al., 2018). Indeed, endogenous CHO stores (e.g., ∼400–750 g muscle glycogen) are often less than the fuel demands of competitive events. Although endurance training increases muscle glycogen storage capacity (Areta & Hopkins, 2018), the main determinants of the muscle glycogen stores are dietary CHO intake and the recovery time since the last fuel‐depleting exercise session (Burke, van Loon, et al., 2017). It is noted that contemporary guidelines for competition preparation no longer promote a ‘high CHO diet’ for all athletes or the complicated classical CHO‐loading protocols involving depletion and loading phases (Karlsson & Saltin, 1971). Rather, a range of targets for CHO intake in the hours (pre‐event meal or between event recovery) to days (general fuelling and glycogen supercompensation) is suggested to allow storage of muscle and liver glycogen commensurate with the fuel needs of the competition bout (see Figure 2). Such strategies, which contribute to ‘high CHO availability’, are quarantined for competition or key training sessions when performance goals are the priority; meanwhile, CHO availability is less important for other training sessions and may even be deliberately reduced for some workouts or phases to enhance metabolic adaptation as part of a plan of periodized CHO availability (Burke et al., 2018).

Contemporary updates on this area of sports nutrition have been made possible by the use of transmission electron microscopy to investigate how subcellular characteristics of storage and utilization of glycogen within the muscle cell explain performance constraints and performance enhancement during endurance exercise (Ørtenblad & Nielsen, 2015), intermittent team sports (Nielsen et al., 2012) and brief supra‐maximal exercise (Gejl et al., 2017). Such studies have shown that the glycogen within muscle cells is stored in three distinct subcellular pools that may have different functions: inter (between)‐myofibrillar, which makes up the largest (∼75%) pool, intramyofibrillar (5–15%) and sub‐sarcolemmal (5–15%) glycogen (Ørtenblad & Nielsen, 2015; Ørtenblad et al., 2013). CHO loading (supercompensation) protocols achieved by higher dietary CHO intake are associated with an increase in the glycogen content of type I fibres, particularly in the subsarcolemmal pool (Jensen et al., 2020, 2021). This is due to an increase in the number rather than the size of existing glycogen particles, ensuring the capacity for rapid rates of utilization (Jensen et al., 2021). Increased storage of intramyofibrillar glycogen content may require the continued stimulus associated with exercise (Jensen et al., 2020). Meanwhile, relative rates of glycogen utilization during endurance cycling appear to be greater in type 1 intramyofibrillar and subsarcolemmal stores and the pre‐exercise intramyofibrillar glycogen content of type 1 fibres is a good predictor of exercise capacity (Jensen et al., 2020). Increased endurance subsequent to CHO loading appears to be associated with a striking sparing of intramyofibrillar glycogen utilization by the above‐normal levels of subsarcolemmal glycogen during the early phase of exercise (Jensen et al., 2020). Glycogen depletion is associated with reductions in sarcoplasmic reticulum vesicle Ca^2+^ release rate and reductions in peak power output (Gejl et al., 2014), and the effect of low muscle glycogen on excitation–contraction coupling may serve as a built‐in mechanism to link the energetic state of the muscle fibre to energy utilization (Ørtenblad & Nielsen, 2015).

Exogenous CHO (CHO consumed in the hours before and/or throughout exercise) can also enhance performance, via a number of centrally and peripherally derived effects including increased neural drive associated with mouth sensing, maintenance of euglycaemia, hepatic and muscle glycogen sparing and the maintenance of high rates of CHO oxidation in the face of dwindling muscle glycogen stores (Karelis et al., 2010; Malone et al., 2021). There is robust evidence that consuming CHO improves performance across a range of sports (Stellingwerff & Cox, 2014; Williams & Rollo, 2015), with a sliding scale of intakes targeting its contribution to muscle fuel needs (Figure 2). Gastric emptying, gastrointestinal comfort and intestinal CHO absorption may be rate limiting for the eventual muscle oxidation of ingested CHO (Malone et al., 2021) and have been the focus of recent activities to address various practical or physiological challenges. Attempted solutions involve ‘training the gut’ by increasing CHO intake in the diet and during exercise to increase tolerance and the activity of sodium‐dependent glucose transporter SGLT‐1 (Costa et al., 2017; Jeukendrup, 2017) and the use of bespoke or novel CHO blends (Baur & Saunders, 2021). The latter include glucose–fructose mixtures that utilize different gut transporters. They can also increase total intestinal absorption and rates of muscle oxidation of ingested CHO (Jeukendrup, 2010), CHO encapsulated with pectin and alginate to form a ‘hydrogel’ (King et al., 2020) and CHO molecules or modified starches with either a fast or slow rate of digestion to match an athlete's opportunities for intake with the ideal fuel availability (Baur & Saunders, 2021). There is often a mismatch between the testimonials for these products and the results of laboratory research. For example, although commercial hydrogel products have been quickly adopted by elite athletes (Sutehall et al., 2018) and publicized as contributing to sporting successes including the 1:59 marathon project (Maurten, 2020), the first wave of studies failed to find evidence of faster gastric emptying, greater gut comfort or muscle fuel delivery or superior performance support (King et al., 2020). In fairness, however, protocols involving elite athletes running at very high absolute and relative speeds have not been sufficiently investigated. Furthermore, several more recent studies have reported some benefits including enhanced gastric emptying at rest (Sutehall et al., 2020), better gut tolerance, CHO oxidation and performance during prolonged exercise (Rowe et al., 2021) and, potentially less dental cariogenicity due to the absence of acidulants (Pettersson et al., 2020) in comparison to traditional CHO drinks.

### 3.3 Strategies to provide alternative oxidative fuels

A countermeasure to strategies that increase CHO availability is to promote other oxidative substrate sources that can extend or replace their finite contribution as muscle fuels. Theoretically, the relatively large lipid stores in even the leanest of athletes represent a potentially unlimited source of fuel for prolonged aerobic exercise. However, unlike CHO oxidation, which is closely geared to the energetic demands of the working muscles, mechanisms to match the availability and metabolism of FA to the prevailing energy expenditure are lacking. Short‐term strategies (e.g., overnight fasting or low CHO, high fat (LCHF) meals in the hours or days before an event) have proved unsuccessful in enhancing performance even in endurance‐trained participants with enhanced capacity for FA oxidation (Burke & Hawley, 2002). Although such strategies increase FA availability, the small increase in FA oxidation is insufficient to replace the contribution of near‐depleted liver and muscle CHO (Burke & Hawley, 2002). However, robust retooling of the muscle to enhance the availability, transport, uptake and utilization of muscle lipids can occur in as little as 5–10 days of adaptation to a LCHF diet involving reduction (∼2.5 g kg^−1^ day^−1^; Burke et al., 2002; Cameron‐Smith et al., 2003) or restriction (<50 g day^−1^; Burke, Whitfield, et al., 2021) of CHO, effectively doubling rates of fat oxidation during exercise and shifting the exercise intensity at which maximal fat oxidation occurs from ∼45% to ∼70% of maximal aerobic capacity (Volek et al., 2016). Despite this, benefits to endurance performance from short (5–10‐day) to medium (∼4‐week) term exposure to non‐ketogenic and ketogenic versions of LCHF diets have been, at best, limited to specific scenarios or individuals (Burke, 2015). Indeed, although such ketogenic LCHF diets have been shown to maintain exercise capacity in some individuals during submaximal (∼60–65% ${\dot{V}}_{O_{2}\mathrm{peak}}$) exercise (Phinney et al., 1983), there is robust evidence of performance impairment during real‐life competition involving higher‐intensity endurance events, which was attributed at least in part to the reduction in exercise economy (increased oxygen cost of exercise) associated with the stoichiometry of fat versus CHO oxidation (Burke, Ross, et al., 2017; Burke et al., 2020).

Strategies to integrate enhanced fat oxidation with a restoration of high CHO availability have been attempted with both ketogenic (Burke, Whitfield, et al., 2021) and non‐ketogenic versions of LCHF diets (Burke et al., 2000, 2002; Carey et al., 2001), without change to the previously observed performance outcomes. Indeed, the continued reduction in exercise CHO utilization (‘glycogen sparing’) seen following the combination of strategies, initially thought to be advantageous in preserving this fuel for later oxidation, was recognized as *impaired* CHO utilization via reduced muscle glycogenolysis and down‐regulation of flux through the citric acid cycle via pyruvate dehydrogenase inactivation (Stellingwerff et al., 2006). Therefore, noting that success in many high‐performance sports is determined by high‐intensity aerobic exercise, maintained throughout the event or at critical stages, the better economy of ATP production from optimized CHO availability/oxidation is most suited to achieve the highest sustainable rates of muscle energy turnover (Burke, 2021). Although more research is needed to investigate the long‐term effects of ketogenic LCHF diets on health and metabolism, the evidence from current performance‐focused studies suggests that they are limited in application to a small range of sporting events in which rates of energy production are low enough to be provided by fat oxidation or in which the athlete is unwilling or unable to support optimal CHO use (Burke, 2021).

The ketone bodies (‘ketones’), acetone, acetoacetate and β‐hydroxybutyrate (βHB) are chemicals produced by the liver during periods of low energy or low CHO availability, with high circulating levels seen during starvation, prolonged fasting and ketogenic LCHF diets. Their contribution to metabolism and performance changes associated with the latter ‘keto‐adaptation’ is unclear from the currently available studies of short to medium LCHF exposure. Indeed, while ketone oxidation is proposed to have an advantage in terms of free energy liberation, it also appears to make only a minor contribution to fuel use during exercise, even when blood concentrations are increased (Dearlove et al., 2021). From a metabolic perspective, βHB is the most important ketone and blood concentrations of 1–3 mmol l^−1^ are considered to be the optimal range for it to exert effects (Evans et al., 2017). It has been recently proposed that oral ketone supplements can be consumed to acutely increase blood concentrations of βHB/ketone without the need to undertake energy or carbohydrate restrictions, to create a unique metabolic state (Dearlove et al., 2021). However, it appears that only ketone esters, particularly the newly commercialized ketone mono‐ester (*R*)‐3‐hydroxybutyl (*R*)‐3‐hydroxy‐butyrate (Cox et al., 2016), are able to practically raise blood ketone concentrations to desired levels, while other forms such as ketone salts or 1,3‐butanediol lack such efficacy. In the decade since this ketone ester became known for its widespread use in the preparation of the British Olympic Team for the 2012 London Olympics and among professional cyclists, nearly 20 studies of the acute intake of various oral ketone supplements have been published. These studies have focused on direct benefits to physiological and cognitive aspects of endurance performance via fuelling mechanisms, although other effects on recovery and training adaptation due to chronic use have also been suggested (Poffé & Hespel, 2020). Two studies have reported performance enhancement associated with the acute use of the ketone ester supplement under specific conditions: prolonged exercise undertaken in overnight fasted subjects (Cox et al., 2016) or with the co‐ingestion of bicarbonate to counter the slight increase in acidosis associated with ketone bodies (Poffe, Ramaekers, et al., 2021). Nevertheless, meta‐analyses of the literature on oral ketone supplements have failed to find clear benefits, noting the large variability in protocols of use (doses, timing of intake and accompaniment with carbohydrate or bicarbonate) and protocols of exercise (Margolis & O'Fallon, 2020; Shaw et al., 2020; Valenzuela et al., 2020). Different protocols of use may achieve variable effects on the timing and increase in blood βHB concentrations with differential effects on substrate metabolism and physiological effects. Challenges with the use of oral ketone supplements include difficulties in pinpointing beneficial uses, the small window of benefit, and the potential for impairment of sports performance via gastrointestinal disturbances (Leckey et al., 2017), altered acid–base balance (Poffe, Ramaekers, et al., 2021) or other effects to be determined (Poffe, Wyns, et al., 2021). A recent study from our group found that the use of a ketone ester supplement in elite race walkers who had undertaken short‐term adaptation to an LCHF diet failed to alter gross measurements of substrate utilization during exercise or attenuate the decline in high‐intensity endurance race performance associated with the LCHF diet (Whitfield et al., 2021).

## 4 STRATEGIES WITH CNS EFFECTS

The role of the brain and CNS in skill‐based sports and events requiring concentration and decision‐making is well known. Only recently, however, have we identified their role in the performance of even simple locomotor events, including strategies around motivation and pacing. Although available research methods have historically favoured the study of peripheral manifestations of a performance decline (Hawley et al., 2015), new strategies in sports nutrition or new insights into the mechanisms underpinning existing strategies have raised the recognition of central issues. Such recognition has often changed protocols of use or increased the range of scenarios to which such strategies or nutritional aids have been directed. The evolution in knowledge and practice around caffeine use in sports performance (summarized in Table 1) has been partly driven by this switch in interest. Caffeine, an adenosine receptor antagonist, has been used as a performance aid in professional sport for more than a century, although anecdotal reports of the use of methyl xanthine sources by ancient workers and warriors to promote endurance pre‐date these accounts. Intriguingly, the 1990s viewpoint on caffeine use in sport, underpinned by decades of focused scientific research, appeared to be largely disconnected from the personal experiences of the majority of adults who consume caffeine regularly to support their sense of well‐being and their capacity to complete activities of daily living (Burke et al., 2013).

**TABLE 1:** Changes in knowledge and practice around caffeine and sports performance

Issue	Caffeine in the 1990s^a–c^	Caffeine in the 2020s^f–k^
Targeted sporting events	Endurance sports	Endurance sports (>90 min)^f^, team^l,m^ and racquet sports^n^, sustained high‐intensity sports of 1–20 min (e.g., rowing^o^ and swimming^p^) and 20–90 min^f^ (e.g., half marathon and cycling time trial), combat sports^q^, power and strength endurance sports^r^, skill sports (e.g., golf^s^, fencing^t^)
Mechanisms of action	Caffeine supplementation is associated with an increase in circulating fatty acids that can create glycogen sparing	Although effects on the body are widespread, beneficial actions of caffeine on sports performance arise primarily via reduction in perceptions of pain, effort and fatigue, attenuating usual decline in performance outcomes within sporting events^f,g^. Direct effects on muscle contractility and force generation are possible, particularly at higher doses^u,v^
Protocol of use	6 mg kg^−1^ taken 60 min prior to exercise	Variety of protocols^f,g^ include single or multiple intakes pre‐ and during sporting events, including just around the onset of the performance decline^w^. Optimal protocols vary with individual and context of use, within range of 3–6 mg kg^−1^; new appreciation of efficacy of doses at low end of range^x^. Some evidence that protocols continue to be effective when use is repeated for multi‐stage sports^y^. Purposeful use of caffeine as a training aid to enhance the quality of workouts, especially under conditions of fatigue^z^
Typical caffeine sources	Laboratory studies: anhydrous caffeine powder. Real world sport use: coffee, cola drinks, caffeine tablets. Some concern that coffee may not be ideal caffeine source due to counteracting effects of other ingredients^d^	Laboratory studies and real world sports use: variety of forms^f,aa^ including caffeinated sports foods (gels, bars), energy drinks^bb^, pre‐workout supplements, coffee, caffeine tablets, caffeinated gum, cola drinks and nasal sprays. Coffee is now considered to be an ergogenic form of caffeineg,^cc^ but may have some practical challenges to achieve a targeted dose due to wide variability in the caffeine content of the same coffee ‘serves’^dd,ee^
Underpinning research	Laboratory based studies, often using protocols of running or cycling *capacity* (e.g., time to exhaustion)	Studies on the performance of a variety of sports, including investigations of real‐world scenarios using high calibre athletes, in a simulated competition in field conditions^ff,gg^ or actual competition^hh,ii^
Personalization of advice/use	Outliers or non‐responders identified in some studies, but caffeine advice typically presented as ‘one size fits all’	Individual differences in response to caffeine recognized, with potential to be at least partially explained by gene polymorphisms, including those involved with hepatic caffeine metabolism (e.g., CYP_1_A_2_) or adenosine receptor activity (e.g., ADORA_2_A)^j,jj,kk^. Further investigation needed to explain apparent contradictions in study findings^j,jj,kk^. Athletes guided to develop practiced and personalized plans for caffeine use according to the context of their sporting goals and individual experience^z^
Integration into athlete's lifestyle	Targeted uses to aid performance in sports competition, usually involving single event use in doses larger than ‘everyday’ use of caffeine. Separate rationale and protocols for ‘social’ or ‘lifestyle’ uses	Athletes guided to consider their performance‐focused use of caffeine within their total diet and lifestyle practices and to periodize its use within their annual training plans^z^. Such integration reduces risk of excessive caffeine use and creates opportunities to trial new competition practices, use caffeine to enhance training quality and adaptation, and include periods to manage habituation to caffeine
Beliefs regarding habitual use and pre‐event caffeine withdrawal	Since habituation to caffeine may lead to reduction in effects, usual protocol adopted in studies and real world use involves pre‐event caffeine withdrawal for 48 h to 7 days	Despite inconsistency in protocols to define habitual intake^ll^, habituation to caffeine reduces but does not negate benefits to performance^mm^, especially if dose is increased above regular intake^j,z^. Short‐term withdrawal before competition or study does not potentiate net benefit but may exaggerate apparent effect if associated with reversal of withdrawal side effects^nn^. Periodic resetting of habitual intake may be useful^z^
Rules regarding use in sport^d^	Considered a banned substance by relevant anti‐doping agencies (International Olympic Committee: 1984 and World Anti‐Doping Agency: 2000) when urine concentrations exceed specified level (15 μg ml^−1^ until 1985 then reduced to 12 μg ml^−1^)^d,e^. Principle: urinary caffeine concentrations can differentiate between purposeful use of caffeine for sports performance and ‘everyday’ use	Removed from WADA Prohibited List. WADA monitoring programme tracks competition use by athletes, albeit with various limitations, by inspecting trends in urinary caffeine concentrations from samples taken at post‐competition anti‐doping control stations. Analysis from 2004 to 2015 suggests that caffeine is used in moderate amounts, prevalence of use and size of doses has increased over time and athletes in endurance sports have highest urinary concentrations^oo^

^a^Graham et al. (1994). ^b^Graham (2001). ^c^Spriet (1995). ^d^Delbeke and Debackere (1984). ^e^Van Thuyne and Delbeke (2006). ^f^Burke (2008). ^g^Guest et al. (2021). ^h^Grgic, Grgic, et al. (2020). ^i^Pickering and Grgic (2019a). ^j^Pickering and Kiely (2019). ^k^Pickering and Grgic (2021). ^l^Salinero et al. (2019). ^m^Ferreira et al. (2021). ^n^Vicente‐Salar et al. (2020). ^o^Grgic, Diaz‐Lara, et al. (2020). ^p^Lara et al. (2015). ^q^Lopez‐Gonzalez et al. (2018). ^r^Grgic et al. (2019). ^s^Mumford et al. (2016). ^t^Bottoms et al. (2013). ^u^Bazzucchi et al. (2011). ^v^Domaszewski et al. (2021). ^w^Cox et al. (2002). ^x^Spriet (2014). ^y^Stadheim et al. (2014). ^z^Pickering and Kiely (2018). ^aa^Wickham and Spriet (2018). ^bb^Souza et al. (2017). ^cc^Pickering and Grgic (2019b). ^dd^Desbrow et al. (2007). ^ee^Desbrow et al. (2019). ^ff^Puente et al. (2017). ^gg^Del Coso et al. (2014). ^hh^Del Coso et al. (2013). ^ii^Portillo et al. (2017). ^jj^Grgic et al. (2021). ^kk^Barreto et al. (2021). ^ll^Filip et al. (2020). ^mm^Lara et al. (2019). ^nn^Irwin et al. (2011). ^oo^Aguilar‐Navarro et al. (2019).

The updated status of caffeine in sports performance integrates this ‘everyday use’ with the specialized needs of athletes. Interest in its putative role in altering exercise metabolism (Spriet et al., 1992) has been superseded by an appreciation of its effects in masking perceptions of effort, pain or fatigue (Spriet, 2014), or its direct effect on the contractility of muscle fibres via changes in calcium handling (Bazzucchi et al., 2011; Domaszewski et al., 2021). Contemporary athlete practices, summarized in Table 1, involve greater sports‐specific uses of caffeine (Burke, 2008), but in smaller doses (Spriet, 2014), from a wider variety of food and supplemental sources (Guest et al., 2021; Wickham & Spriet, 2018), in a greater variety of protocols of intake around an exercise session (Burke, 2008), with better integration or periodization into daily dietary and lifestyle practices (Pickering & Grgic, 2021) and with consideration of individual responsiveness (Pickering & Kiely, 2018; Southward et al., 2018). Indeed, the richness of the recent literature has allowed an umbrella analysis of 21 previous meta‐analyses (Grgic, Grgic, et al., 2020), drilling down to meta‐analyses of its effects on groups of sports (e.g., team sports; Salinero et al., 2019) and even further to specific events (e.g., soccer; Mielgo‐Ayuso, Calleja‐Gonzalez, Del Coso, et al., 2019), sex (Mielgo‐Ayuso, Marques‐Jiménez, et al., 2019) and forms of intake (e.g., energy drinks (Souza et al., 2017) or coffee (Pickering & Grgic, 2019b)). Further innovation in future practices may come from better knowledge about the effects of training status, sex, habitual caffeine intake, genetics and circadian influences on responsiveness to performance outcomes (Martins et al., 2020; Pickering & Grgic, 2019a). Interrogation of practical issues such as repeated use for multi‐stage competitions and interaction with other competition nutritional strategies is also of merit (Burke, 2017).

Another evolving theme around nutritional strategies to enhance the CNS contribution to sports performance involves food‐derived factors and properties that do not even need to be absorbed into the body to achieve beneficial effects (Best et al., 2021; Burke & Maughan, 2015). Sensory input from taste receptors in the mouth and upper digestive tract, and perhaps olfactory and other sensory cues, act to encourage our intake as well as to commence the processes of digestion and absorption (Breslin, 2013). This interest first emerged in sports nutrition when it was recognized that exogenous CHO intake could improve ∼1‐h cycling time trial performance, even though muscle substrate (glycogen) availability is not rate limiting for this event (Jeukendrup et al., 1997). Such performance benefits were not achieved by the intravenous delivery of glucose to the muscle (Carter, Jeukendrup, Mann, et al., 2004), but were detected when the mouth was simply rinsed with a glucose or maltodextrin solution (Carter, Jeukendrup, & Jones, 2004) exposing receptors in the oral cavity to CHO and stimulating reward centres in the brain to increase pace or work output (Chambers et al., 2009). Benefits seem to be achieved by frequent (every 5−10 min) and significant (10 s) contact between the oral cavity and a carbohydrate source (Burke & Maughan, 2015; Jeukendrup & Chambers, 2010), independent of a sweet taste (Chambers et al., 2009), and are accentuated under conditions of low CHO availability such as an overnight fast or low muscle glycogen (Lane et al., 2013). Although this science is still in its infancy, there is evidence that the exposure of the oral cavity and gut to nutrients, components or characteristics of food/fluids that activate regions or responses in the CNS can enhance pacing, reduce the perception of effort or increase corticomotor activity to enhance performance of a variety of sports (Best et al., 2021). Table 2 (Burke & Maughan, 2015) summarizes current knowledge around the pre‐absorptive responses to the presence of such compounds/features.

**TABLE 2:** Summary of tastants, components and characteristics of foods and fluids that may achieve central nervous system effects via ‘mouth sensing'^a,b^

Component	Summary of mouth sensing effect	Mechanism and protocols of use	Potential scenarios of use (requiring individualization and practice)
Carbohydrate^c–f^	Strong evidence that mouth sensing of CHO interacts with CNS to enhance perception of well‐being and ‘energy’, promoting increase in pacing (e.g., increased speed, power). Performance benefits seen in well‐tested models (males, sustained exercise) but need to be further extended to others (e.g., elite, females, intermittent sports, field conditions)	Single and serial (e.g., 5–10 min) swilling of CHO (25 ml dose), to achieve 5–10 s exposure of receptors in mouth, stimulates brain centres involved in reward and motor control. Effect is achieved by CHO rather than sweet taste. Effect does not require swallowing of CHO. Effect is enhanced in scenarios of low CHO availability (e.g., overnight fasted, glycogen depleted)	Good evidence/theory for several applications: non‐endurance events: 45–75 min. Additional performance benefit for fuelling strategies in endurance events >90 min. Short‐term alternative if gut discomfort prevents CHO ingestion during parts of endurance event. Attenuation of performance decrement when deliberately training in fasted state or with low CHO availability
Fluid^g–i^	Although it has not been fully and directly investigated, indirect evidence from studies of different modes of fluid delivery during exercise (intravenous, mouth swilling without swallowing, nasogastric delivery and swallowing fluid) suggests that the swallowing of fluid causes interaction with oral‐pharyngeal receptors that may affect thirst, fluid regulation and performance. It is currently unknown if or how such interaction might be accentuated to enhance performance effects of fluid intake during exercise		
Bitter (quinine)^j–l^	Preliminary evidence that mouth sensing of quinine, a bitter compound, may stimulate an immediate enhancement of brief maximal/supra‐maximal exercise akin to ‘fight or flight’ response	Swilling (10 s) and swallowing of 25 ml of 2 mmol l^−1^ quinine solution immediately prior to maximal exercise task may activate bitter (T2R) receptors in back of oral cavity and upper GI tract to stimulate sympathetic nervous system responses and/or corticomotor excitability. Effect not achieved unless quinine is swallowed suggesting adequate exposure of receptors in throat and upper GI tract is important. Effect seems to occur despite individual differences in bitter taste sensitivity	Some evidence of benefits to single brief maximal/supramaximal event. Unclear if benefits are seen with sequential use (e.g., prior to repeated sprint efforts in longer event) or single use just prior to the final effort
Caffeine^m,n^ (bitter?)	Unclear evidence that mouth rinsing with caffeine solutions may create an immediate enhancement of performance, possibly via its bitter taste. Lack of clarity due to heterogeneity of current study protocols, including failure to swallow mouth rinse	Although some absorption of caffeine may occur via oral buccal cells, potential effect of mouth sensing of caffeine solutions may occur via activation of bitter T2R receptors. Effect not seen unless caffeine mouth rinse is swallowed suggesting importance of adequate exposure to receptors in throat and upper GI tract. Suggested that these receptors could be activated even if form of caffeine in sports products is sweet	Further investigation needed, but may have application: immediately prior to brief maximal/supra‐maximal exercise tasks; single or serial application within longer exercise tasks prior to critical effort(s)
Cool (menthol)^o–q^	Good evidence that menthol, particularly the l‐isoform, creates a perception of cooling when exposed to skin and mouth to increase pacing (increased power, speed, etc.) during prolonged exercise in heat. Effect best seen with internal ingestion, including mouth rinse suggesting importance of sensors in mouth. Performance benefits seen in well‐tested models (males, sustained exercise in laboratory) but need to be further extended to others (e.g., females, intermittent sports, field conditions)	Single and serial (every 5–10 min) mouth rinsing with 25 ml l‐menthol containing solutions activates TRPM‐8 channel sensors in oral cavity to reduce improve thermal sensation/comfort and increase pace during continuous exercise. May also increase nasal patency and ventilation	Good evidence for application of single or serial use of menthol mouth rinse: continuous exercise in hot environments >3 min. Effects seen with work output but further studies needed to investigate all performance outputs (e.g., skills) and event types (e.g., intermittent, team)
‘Anti‐cramp agents'^r–t^: acetic acid (pickle juice), capsaicin (capsicum), cinnamaldehyde (cinnamon) and ginger	Anecdotal and laboratory based evidence of small reductions in the risk and severity of muscle cramps following intake of several plant‐based compounds associated with hot or spicy tastes	Some evidence from laboratory‐invoked cramp models that mouth swilling and swallowing of pickle juice or combination of capsaicin, cinnamaldehyde and ginger can temporarily (30 s–15 min) reduce susceptibility or characteristics of muscle cramps. Transient receptor potential channels in the oropharyngeal region, associated with transduction of spicy or hot tastes (e.g., TRPV1 and TRPA1 channels), are stimulated by exposure to these compounds. The burst in activity may attenuate the hyperexcitability of α‐motor neurons potentially involved with exercise‐associated muscle cramps. Further investigation of application to field conditions is required	Initial evidence that mouth rinse with ‘anti‐cramp agents’ may reduce risk and severity of exercise‐associated muscle cramps in susceptible athletes warrants further investigation

^a^Burke and Maughan (2015). ^b^Best et al. (2021). ^c^Chambers et al. (2009). ^d^Jeukendrup (2013). ^e^Pochmuller et al. (2016). ^f^de Ataide e Silva et al. (2013). ^g^James et al. (2019). ^h^Armstrong and Kavouras (2019). ^i^Arnaoutis et al. (2012). ^j^Gam et al. (2016). ^k^Etxebarria et al. (2021). ^l^Etxebarria et al. (2019). ^m^Pickering (2019). ^n^Ehlert et al. (2020). ^o^Barwood et al. (2020). ^p^Stevens and Best (2017). ^q^Jeffries and Waldron (2019). ^r^Miller et al. (2010).

^s^Craighead et al. (2017). ^t^Behringer et al. (2017). Abbreviations: CNS, central nervous system; GI, gastrointestinal; TRPM‐8, transient receptor potential melastatin type 8.

This knowledge can lead to a number of changes to the practices of athletes. First, it increases the opportunities for meaningful nutrition strategies immediately before or during sports that are too short to allow nutrient absorption or were previously considered too brief to require nutrient support. Second, it contributes a separate and additional mechanism by which nutrients consumed during prolonged events to provide whole body or peripheral benefits (e.g., fluid, CHO) can enhance performance; here, it may alter traditional strategies of timing and sources of intake of these nutrients during competitive events to optimize their CNS benefits (Burke & Maughan, 2015). Finally, it offers an alternative strategy in scenarios when an athlete may be unable to ingest/swallow foods and fluids. Although some tastants may need to be swallowed to allow contact with receptors that are largely located in the back of the throat and upper gastrointestinal tract, in some cases, the athlete may be able to gain a substantial performance boost simply by swilling and expectorating the product. This might provide performance strategies to athletes who undertake events during periods of fasting (e.g., Ramadan), those who include periodic workouts with low CHO availability for enhanced training adaptation or energy restriction for body composition goals (Burke et al., 2018), or if gastrointestinal discomfort/upsets during exercise prevent CHO ingestion (Burke & Maughan, 2015). While the performance outcome may be less than if nutrients were consumed according to recommendations, it may still be superior to the absence of any strategy (Lane et al., 2013). One caveat to the use of mouth sensing nutritional tactics is that the athlete may misjudge their overall event pacing; a psychological sensation of being fuelled, cool or well‐hydrated in the absence of the physiological benefit may cause the athlete to increase work output to a non‐sustainable pace that may actually lead to premature onset of a performance decline and other concerns related to hyperthermia (Barwood et al., 2020; Stevens et al., 2018). Individualization and practice are key to developing successful methods, while innovation may be needed to achieve the logistics around mouth/gut exposure to these compounds and characteristics in various sporting events.

## 5 FLUID REPLACEMENT DURING COMPETITION – NEW THOUGHTS ON AN OLD BATTLE

During many sporting events, the evaporation of sweat plays a substantial role in the dissipation of excess heat produced by the working muscle or absorbed from the environment, reducing body water stores in the process (Gonzalez et al., 2009; Periard et al., 2021). Athletes can counter sweat losses by consuming fluid during the event (Garth & Burke, 2013) or, in a lesser contribution, by hyperhydrating in the hours pre‐event via ingestion of large volumes of fluid containing osmolytes such as glycerol and/or sodium chloride (Goulet et al., 2018). However, in the majority of situations in high‐performance sport, the opportunity or desire to drink fails to keep pace with sweat losses, leading to a body fluid deficit (Garth & Burke, 2013). Furthermore, some athletes may commence the event with pre‐existing hypohydration, often due to the failure to reverse the fluid deficit from a prior exercise bout or the deliberate dehydration strategies associated with ‘making weight’ for weight category sports (Burke, Slater, et al., 2021). Sweat rates during sports competitions vary markedly according to the type and intensity of the event, the environmental conditions in which it occurs (temperature, humidity, wind speed, altitude, etc.), the size and body composition of the athlete, their clothing or other protective gear and their degree of training status and acclimatization (Gonzalez et al., 2009). Prediction equations can estimate sweat losses associated with many of these variables (Gonzalez et al., 2009), but are generally unable to account for the complexity of the interaction of these factors in real life sporting competitions (Jay & Webb, 2009).

Despite more than 50 years of research on hydration for sporting events, two separate but related issues still create remarkable contention: (When) does hypohydration impair sports performance? How should athletes approach hydration strategies for their event? Since sweat is typically hypotonic with respect to plasma, exercise‐associated sweat losses lead to hypertonic hypovolaemia (Sawka et al., 2015). Reduced plasma volume is associated with increased cardiovascular strain, an elevated perception of effort, and a decrease in muscle blood flow and aerobic reserve, particularly in hot conditions where the competition between peripheral and central circulation is exacerbated by the redistribution of blood to cutaneous vascular beds to dissipate heat. There is an increase in heat storage as sweat rates for any given core temperature are reduced, particularly as plasma osmolality increases. Other factors associated with hypohydration, particularly when exercise is undertaken in a warm to hot environment, include increases in muscle glycogen utilization, motor unit recruitment and afferent feedback, skin temperature, thermal discomfort and thirst‐derived distraction (for reviews, see Cheuvront et al., 2010; Periard et al., 2021; Sawka et al., 2015). The physiological and psychological responses to hypohydration are a continuum, with the magnitude of impairment being linearly related to the magnitude of the fluid deficit, and exacerbated with environmental stress (Montain & Coyle, 1992).

Although these effects are well‐characterized, their translation to competition outcomes in high‐performance sports is controversial (Cotter et al., 2014; Sawka & Noakes, 2007). Indeed, the real‐life outcome will be mediated by a complex interaction of factors around the individual athlete, the type of event, the environmental conditions in which it is conducted, the importance of absolute versus relative performance (i.e., does the athlete need to perform optimally or just better than other competitors?), the size of the fluid deficit and whether it was present at the start of competition or accumulated over the event. Recent summaries of the mostly laboratory‐based literature on hypohydration and exercise performance show some discrepancies, but also the likelihood that at some point, hypohydration will impair performance to a degree that is meaningful to competitive success (Cheuvront & Kenefick, 2014; Goulet, 2013; Judelson et al., 2007; Nuccio et al., 2017; Savoie et al., 2015). According to these reviews, scenarios of greatest risk are prolonged submaximal/intermittent exercise undertaken in hot conditions and when skin temperature is raised, when the fluid deficit exceeds 3% BM and, perhaps, when there is an overlay of skill and cognitive performance. Flaws in the application of laboratory studies of hydration and performance include their lack of integration of the environmental conditions, motivational incentives and success determinants of real‐life sport as well as their failure to capture the timing and magnitude of hypohydration to which athletes are commonly exposed (Cotter et al., 2014; Maughan, 2012). Factors that suggest that the effects of hypohydration are overstated include the inability to replicate the motivational aspects of real‐life competition that might elicit extra effort to counter the effects of hypohydration or consideration from studies and anecdotal observations that successful athletes can tolerate high levels of hypohydration and that repeated exposure to hypohydration may lessen its effects (Burke, 2019). The lack of blinding and potential placebo/nocebo effects may also present in a large majority of hypohydration studies, distorting the physiological effects of dehydration or the effects of oral intake of fluid on performance.

Countering this, it is noted that studies that examine performance of whole exercise task while hypohydration only reaches critical threshold later in exercise may fail to observe impairments at the critical stage of an event, while the magnitude of performance change required for significance in a study may underestimate the tiny margins that determine success in real life (Burke, 2019). Protocols that are better able to target effects of hypohydration on sports performance should include features such as the removal of the placebo and sensory effects of drinking fluid (James et al., 2019). This has been achieved in some recent studies by oral intake of small amounts of fluid to provide identical sensory exposure in each trial, while manipulating hydration via the blinded infusion of different volumes of fluid intake into the stomach with a nasogastric tube (Funnell et al., 2019; James et al., 2017).

Guidelines for fluid intake during sporting competitions have evolved markedly but contentiously over the past decades. Hallmarks have included recommendations to relax historical restrictions on fluid intake within official race rules (American College of Sports Medicine, 1975), pro‐active but formulaic guidelines for intake of fluid with the goal of minimizing the mismatch between fluid intake and sweat losses (American College of Sports Medicine, 1987), and revised positions for individualized ‘programmed’ fluid intake plans that defend (where possible) a gold standard of hydration (suggested as loss of <2% BM over the event) but warn against over‐drinking (shown by a gain in BM) to prevent the development of hyponatraemia (Sawka et al., 2007). However, even the latter viewpoint has attracted harsh criticism that humans need only drink fluids ‘to thirst’ or ‘ad libitum’ during exercise/sporting activities and that opinions to the contrary represent flawed research supported by biased sports scientists and commercial interest in the sale of sports drinks (Noakes, 2012; Noakes & Speedy, 2006). Indeed, the current situation presents a challenge to contemporary sports scientists and athletes to choose between two camps promoting apparently opposite approaches (programmed/planned drinking vs. drinking to thirst/ad libitum drinking). Even recent attempts to present a unification of these polarized themes (Kenefick, 2018) have received rapid opposition (Goulet, 2018; Valenzuela et al., 2018), arguably with overstatement of the actual differences in position (Kenefick, 2019).

**FIGURE 3:**
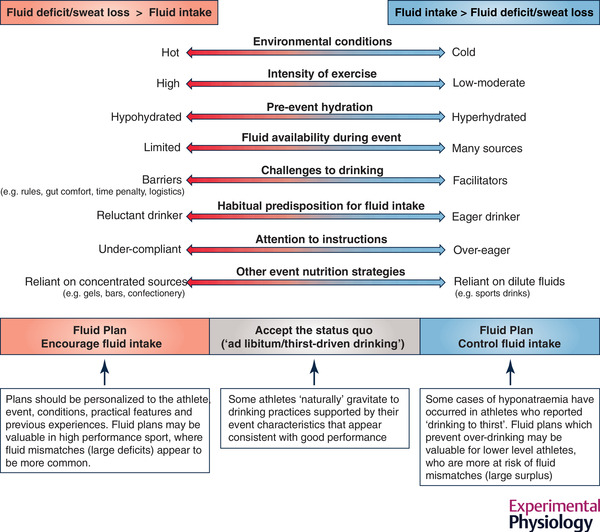
Integrated model of guidelines for fluid intake during sporting competitions. A range of factors related to the exercise task, the athlete and the environment interact to create the direction and magnitude of the match between sweat losses and the athlete's fluid intake. In some scenarios, the resulting fluid balance is compatible with performance and safety; the athlete can continue to follow their usual/‘natural’ hydration practices. In other scenarios where there is a large mismatch, individualized hydration plans should either encourage fluid intake to reduce the risk of a large fluid deficit or prevent over‐drinking to reduce the risk of a large fluid surplus

This author promotes the theory and practice of a middle ground: supporting the benefits of drinking during competitive sporting events while warning against fluid intake in excess of sweat losses or the total body water deficit that can lead to small but concerning number of cases of morbidity and mortality from cerebral oedema associated with hyponatraemia (Hew‐Butler et al., 2015). However, the route to achieving this is multifactorial because of the previously noted variability in conditions that increase sweat losses and others that promote or impede fluid intake (Burke, 2019). It is beyond the scope of the present review to explore the physiological underpinning of ad libitum or thirst‐driven fluid intake (Armstrong et al., 2020), and whether the behaviour is actually possible during sports competitions, due to the overlay of rules and logistical factors that influence the opportunities to consume fluid. However, a concept is proposed where hydration practices during competitive sport can be viewed, for an individual or for all athletes, in terms of the balance between the accrued fluid deficit and fluid intake that typically occurs based on observation or theoretical calculations (Figure 3). Here, zones can be identified in which the balance of factors typically creates a scenario in which an ad hoc outcome – whether termed as ad libitum or drinking to thirst – is suitable, since health and performance outcomes appear to be commensurate with the efforts associated with fluid intake.

Meanwhile there are zones in which the likelihood of a sub‐optimal balance suggests that better outcomes would be achieved with pro‐active change (a ‘fluid plan’). Indeed, it is noted that case histories of hyponatraemia have included athletes who claimed to be following thirst‐driven drinking practices (Hew‐Butler et al., 2015), suggesting that planned fluid intake for some individuals might include strategies to reduce intake/prevent over‐drinking. However, there are also scenarios, often found in high‐performance sports, in which predictions or experiences confirm the likelihood of accruing a large fluid deficit and suggest that the athlete would benefit from a deliberate and personalized plan to increase fluid intake. Real‐world examples include the high prevalence (93%) of self‐reported use of personalized experience‐based fluid plans by elite distance runners and race walkers during the extreme heat experienced at the 2019 Doha World Athletics Championships (Racinais et al., 2021); implementation of an increased frequency of aid stations (from every 5 to 2.5 km) by organizers of some elite marathon events to facilitate in‐race nutrition support towards faster finishing times (Burke, Jeukendrup, et al., 2019) and the strategy of unhampered access to nutrition support (feeds provided by an accompanying bike rider) during the 2019 successful bid to break the 2 h marathon (albeit unrecognized as an official world record due to the non‐adherence to World Athletics race rules) (Burgess, 2019). This model of a unified approach to fluid intake during sports competition should enable sports scientists, coaches and athletes to learn from published data and personal experience to identify the scenarios in which their events might require different – for example, ad hoc or planned – approaches to hydration.

## 6 SUMMARY

High‐performance sport challenges the athlete to counter the limitations to their ability to be swifter, higher and/or stronger across the duration of their sporting event. Sports performance involves a complex interaction between physiological, biomechanical and psychological factors, with some of factors that cause a decrement in outputs being underpinned by nutritional issues. Dietary strategies undertaken in training or in the immediate period before, during or between competitive events can reduce or delay the onset of such performance constraints, leading to enhanced performance. The science and practice of sports nutrition continues to evolve and includes strategies to provide an ongoing supply of key fuels, to promote CNS approaches to enhanced pacing and to solve differences in the interpretation of data and observations around hydration and performance. Although there is a need for a sound understanding of the theoretical basis of exercise capacity, nutritional strategies need to be developed to suit the logistical features of sporting competitions. Future endeavours should focus on better understanding the interaction of different nutritional strategies within the same event, individual differences in response to nutrition strategies and the athlete's capacity to implement strategies in real‐life conditions including harsh environments and multi‐day or multi‐event programmes.

## COMPETING INTERESTS

None declared.

## AUTHOR CONTRIBUTIONS

Sole author.
